# Primary acinic cell carcinoma of the trachea: A case report and literature review

**DOI:** 10.1097/MD.0000000000032871

**Published:** 2023-02-10

**Authors:** Mai-Qing Yang, Zhi-Qiang Wang, Xiu-Feng Li, Li-Qian Chen, Hai-Ning Zhang, Ke-Xin Zhang, Hong-Tao Xu

**Affiliations:** a Department of Pathology, Weifang People’s Hospital (First Affiliated Hospital of Weifang Medical University), Weifang, Shandong Province, China; b Department of Orthopedics and Trauma, Weifang People’s Hospital (First Affiliated Hospital of Weifang Medical University), Weifang, Shandong Province, China; c Department of Pathology, The First Hospital and College of Basic Medical Sciences, China Medical University, Shenyang, Liaoning Province, China.

**Keywords:** Acinic cell carcinoma, immunohistochemistry, lung neoplasm, morphology, trachea

## Abstract

Salivary gland-type acinic cell carcinoma (ACC) is a low-grade malignancy. Primary ACC of the trachea and lungs is rare; here, we describe 1 such case. The histological morphology of tracheal ACC was similar to that of its salivary gland-associated equivalent. Because of its rarity, it is easily misdiagnosed as another type of tracheal or lung tumor. Microscopic analysis of pathological features and immunohistochemistry help diagnose primary ACC of the trachea and lungs.

**Patient concerns:**

A 33-year-old female complained of shortness of breath and hemoptysis for 2 years, and reported the symptoms to have aggravated over the last 4 months. The patient was admitted to our hospital for further treatment. Enhanced computed tomography revealed a soft tissue density nodule shadow in the trachea, which was approximately 1.3 × 1.2 cm in size.

**Diagnoses:**

Based on the clinical information, morphological features, and immunohistochemistry, the pathological diagnosis was primary ACC of the trachea.

**Intervention:**

The tracheal lesion was resected with an electric snare, electrotomy, freezing, and an argon knife using a rigid bronchoscope.

**Outcomes:**

The patient’s postoperative course was uneventful.

**Lessons:**

It is important to prevent misdiagnosis of this type of tumor as another type of lung tumor. Morphological and immunohistochemical features can be useful in diagnosing primary ACC of the trachea and lungs.

## 1. Introduction

Salivary gland-type acinic cell carcinoma (ACC) is a relatively low grade malignancy.^[1]^ It usually occurs in the salivary glands of the head and neck region, particularly in the parotid gland. Apart from the salivary gland, ACC can also occur in the trachea, lungs, mammary gland, and other organs among which primary ACC of the trachea and lung is very rare.^[1–3]^

## 2. Case presentation

### 2.1. Ethical approval

This study was approved by the Institutional Review Board of China Medical University for Human Studies. The ethical board approval number is LS[2021]009. Written informed consent was obtained from the patient for publication of this case report and accompanying images. This study was conducted in accordance with the principles of the Declaration of Helsinki.

### 2.2. Clinical history

**Figure 1.**
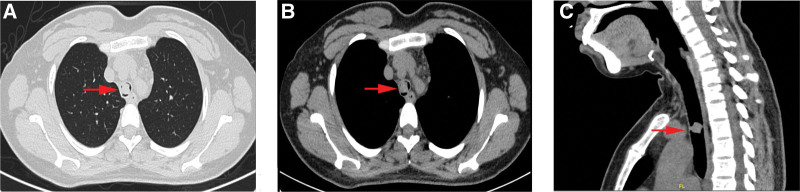
Computed tomography (CT) scan of the chest. Enhanced CT scans revealed a soft tissue density nodule shadow in the tracheal cavity. The size of the lesion was about 1.3 × 1.2 cm with a CT value of about 57 HU. The tracheal lumen was narrow and the tracheal ring was complete. The remaining bronchi were unobstructed, without expansion or stenosis. CT = computed tomography.

A 33-year-old female complained of shortness of breath, coughing, purulent yellow sputum, and bloody sputum 2 years prior to presentation. Four months prior to presentation, the symptoms worsened, including shortness of breath, fatigue, coughing, excessive yellow sputum production, and massive hemoptysis. The patient had no fever, chest pain, or other relevant symptoms. The patient was admitted to another hospital 6 days prior to presentation and received pharmaceuticals for symptomatic treatment, such as “cefodizime,” “ipratropium bromide,” and “N-acetylcysteine.” The patient’s symptoms were not significantly relieved. She was admitted to our hospital for further treatment. Enhanced computed tomography revealed a soft tissue density nodule shadow in the tracheal cavity. The size of the lesion was about 1.3 × 1.2 cm with a computed tomography value of about 57 HU. The tracheal lumen was narrow and the tracheal ring was complete. The remaining bronchi were unobstructed, without expansion or stenosis. The tracheobronchial and subcarinal lymph nodes were not enlarged (Fig. 1). The preoperative diagnosis was intratracheal mass. Resection of the tracheal lesion was then performed using a rigid bronchoscope. During surgery, a tumor mass was observed to protrude into the lumen of the trachea, 6 cm below the glottis, and the lumen was narrow. The tumor was rough and uneven on the surface, with abundant blood supply. The tumor was completely removed with an electric snare, electrotomy, freezing, and an argon knife. The patient did not receive postoperative radiotherapy or chemotherapy and recovered well after surgery. Follow-up at 13 months showed no evidence of recurrence or other metastatic diseases.

### 2.3. Immunohistochemical and histochemical staining

The resected specimens were fixed with 10% neutral-buffered formalin, embedded in paraffin blocks, and cut into 4 μm thick serial sections. The sections were stained with hematoxylin and eosin. Some tumor sections were immunostained with ready-to-use primary antibodies against broad-spectrum cytokeratin (CK), vimentin, CD117, CK5/6, CK7, P40, P63, paired box gene 8 (PAX8), SRY-related HMG-box 10, thyroid transcription factor-1, CD56, napsin A, synaptophysin, S-100, and Ki-67 (Maixin, Fuzhou, China). Immunohistochemistry was performed using EnVision. Tumor sections were also stained with periodic acid-Schiff (PAS) and Alcian blue-periodic acid-Schiff.

### 2.4. Morphological and immunohistochemical findings

**Figure 2.**
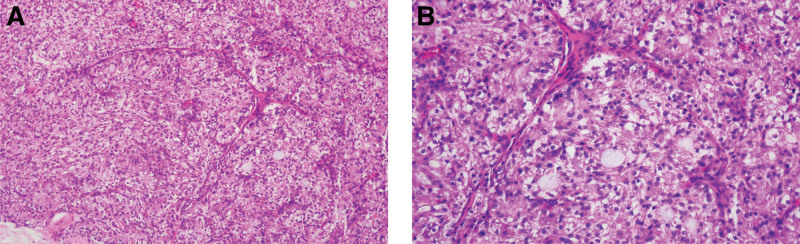
Histological features of primary tracheal acinar cell carcinoma. A. The tumor cells formed solid cell nests, as well as acinar or microcystic structures. Fibrous stroma cells were very few, and blood vessels were thin and abundant (hematoxylin and eosin [H&E] staining, 100 × magnification). B. The tumor cells were relatively uniform, round, or polygonal in shape, with abundant granular eosinophilic or clear cytoplasm. Mucinous secretions were also observed in some tumor cells. The cell nuclei were small, uniform, and bland with fine chromatin. Nucleoli and mitotic figures were not observed (H&E staining, 200 × magnification).

Microscopically, the tumor tissue was located under the bronchial mucosa and exhibited no morphological transition to the bronchial epithelia. The tumor cells formed solid cell nests and acinar or microcystic structures (Fig. 2). There was very little fibrous stroma, and the blood vessels were thin and abundant. The tumor cells were relatively uniform, round or polygonal in shape, with abundant granular eosinophilic or clear cytoplasm. Mucinous secretions have also been observed for some tumor cells. The cell nuclei were small, uniform, and bland with fine chromatin. Nucleoli and mitotic figures were not observed. There were no signs of lymphatic, vascular, or neural invasion.

**Figure 3.**
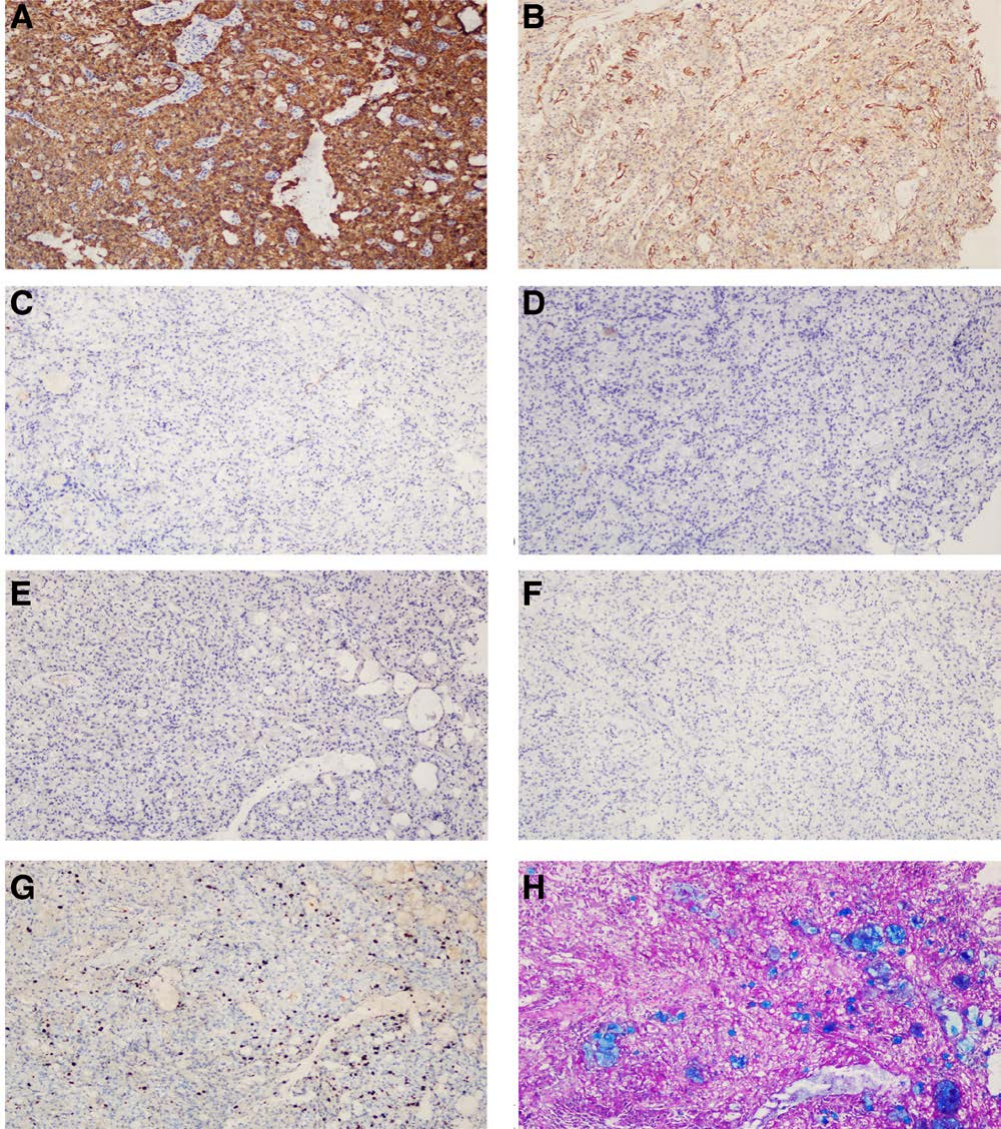
Immunohistochemical and Alcian blue-periodic acid-Schiff staining of primary tracheal acinar cell carcinoma. A. Tumor cells were positive for cytokeratin 7 (100 × magnification). B. Tumor cells were moderately positive for Dog-1. C-F. Tumor cells were negative for S-100 (C), TTF-1 (D), P63 (E), and SOX-10 (F) (100 × magnification). G. The Ki-67 index of the tumor cells was about 5% (100 × magnification). F. The cytoplasm of the tumor cells stained positive with periodic acid-Schiff staining, and the mucinous secretions of some tumor cells stained positive with Alcian blue staining (100 × magnification). SOX-10 = SRY-related HMG-box 10, TTF-1 = thyroid transcription factor 1.

Immunohistochemically, the tumor cells were strongly and diffusely positive for CK and CK7, moderately positive for Dog-1, and negative for vimentin, P63, P40, napsin A, thyroid transcription factor 1, chromogranin A, CD56, synaptophysin, S-100, SRY-related HMG-box 10, and PAX8. The Ki-67 index was approximately 5%. PAS and Alcian blue-periodic acid-Schiff staining results were positive (Fig. 3).

## 3. Discussion

Based on the clinical information, morphological features, and immunohistochemistry results described above, the tumor was diagnosed as primary ACC of the trachea. Primary ACC of the trachea and lungs usually presents as an endobronchial mass because it originates from the submucosal glands. Primary salivary gland-type tumors of the lungs are rare, constituting < 1% of all pulmonary tumors. Primary lung ACC is a low-grade malignant tumor that usually presents as an isolated tumor adjacent to or close to the bronchus.^[1,4]^ In 1972, Fechner et al first reported a case of primary ACC of the lung.^[5]^ To the best of our knowledge, including the present case, only 32 patients with primary ACC of the lung or trachea have been reported so far.^[5–26]^ Clinicopathological features of the patients are summarized in Table 1. The most common symptoms were hemoptysis and coughing, but 1-third (11/32) of the patients were asymptomatic and the tumor was identified incidentally.^[5–26]^ Of the patients with primary ACC of the lungs, 14 were female and 17 were male. The patient age varied widely between 4 to 75 years (mean age: 43.4 years). The diameter of the tumors ranged from 0.4 to 8.6 cm (mean diameter: 2.6 cm). Twenty cases occurred in the right lung, 7 in the left lung, and 5 in the trachea. Two cases of lymph node metastasis have been reported.^[12,13]^

**Table 1:** Clinicopathological characteristics of patients with primary acinic cell carcinoma of the lung and trachea.

Case	Symptoms	Yr	Sex	Age	Size (cm)	Site	Therapy	Outcome
1^[5]^	Asymptomatic	1972	M	63	4.2	RLL	S	A, 22 mo
2^[6]^	Recurrent pulmonary infections	1976	F	12	0.8	RML, bronchus intermedius	S	A, 1 yr
3^[7]^	Three-year history of hemoptysis	1982	M	54	2.2	Trachea	S	A, 2 yr
4^[8]^	Persistent cough	1985	M	36	4	RML	Unknown	Unknown
5^[9]^	Incidental	1992	F	44	3.5	RML, subpleural	S	A, 10 yr
6^[9]^	Incidental	1992	F	48	4	LUL, parenchymal	S	LF
7^[9]^	Incidental	1992	F	50	1.7	RML, subpleural	S	A, 3 yr
8^[9]^	Persistent cough	1992	M	63	1.2	RML, endobronchial	S	A, 4 yr
9^[9]^	Incidental	1992	F	75	1.5	RUL, subpleural	S	A, 4 yr
10^[10]^	Dyspnea, cough, stridor	1994	M	31	2	Trachea	S	A, 8 yr
11^[11]^	Hemoptysis	1996	M	59	3	Trachea	S	ND
12^[12]^	Incidental	1999	M	64	3	LLL, endobronchial	S, radiation	A, 1.5 yr
13^[13]^	Incidental	2003	F	30	2	RLL, subpleural	S	R, 20 mo
14^[14]^	Incidental	2003	M	70	4	RLL, intraparenchymal	S	ND
15^[15]^	Hemoptysis and night sweats	2004	F	4	3	LUL, endobronchial	S	ND
16^[16]^	Hoarseness	2004	M	54	5	Trachea	S	A, 23 mo
17^[17]^	Persistent cough	2004	M	58	1.5	RML, endobronchial	S	A, 4 mo
18^[18]^	Incidental	2005	F	37	3.3	RUL	S	ND
19^[19]^	Incidental	2006	M	47	1.8	LLL, endobronchial	S	A, 1 yr
20^[20]^	Asymptomatic	2010	M	68	1.8	LLL	S, chemotherapy	ND
21^[21]^	Incidental	2011	M	63	5.2	RUL	S	A
22^[22]^	Persistent cough	2011	F	55	1.3	RUL	S, chemotherapy	A, 3 yr
23^[23]^	Persistent cough	2017	M	31	4.5	RLL, subpleural	S	ND
24^[24]^	Persistent cough, hemoptysis	2019	F	10	1.5	LUL	S	A, 30 mo
25^[24]^	Persistent cough, chest pain	2019	M	25	2.2	RUL	S	A, 48 mo
26^[24]^	Hemoptysis	2019	M	37	1	RLL	S	A, 8 mo
27^[24]^	Persistent cough, hemoptysis	2019	F	8	0.4	LML	S	A, 33 mo
28^[24]^	Persistent cough, hemoptysis	2019	M	28	1	RML	S	A, 31 mo
29^[24]^	Hemoptysis	2019	F	53	1.5	RML	S	A, 28 mo
30^[25]^	Persistent cough, hemoptysis	2021	F	27	8.6	RUL, endobronchial	S	A, 24 mo
31^[26]^	Persistent cough, hemoptysis	2021	M	53	2.2	RUL, endobronchial	S, radiation	ND
32 (present case)	Hemoptysis	2022	F	33	1.2	Trachea	S	A, 13mo

A = alive, F = female, LF = lost to follow-up, LLL = left lower lobe, LUL = left upper lobe, M = male, ND = not described, RLL = right lower lobe, RML = right middle lobe, RUL = right upper lobe, R = recurrence.

The histological features of this type of tumor are extremely similar to those of ACC of the salivary glands in the head and neck region. Primary ACC of the lung or trachea may be confused with other primary or metastatic lung tumors. First, metastatic acinar cell carcinoma of the head and neck should be excluded through detailed medical history and clinical examinations. Some tumors with similar histological characteristics need to be distinguished from primary pulmonary ACC, such as mucoepidermoid carcinoma, pulmonary adenocarcinoma, squamous cell carcinoma, carcinoid tumor with a clear or eosinophilic cell component, and metastatic renal clear cell carcinoma. Mucoepidermoid carcinoma usually features 3 distinct cell types: mucin-secreting, intermediate, and squamoid cells. Intermediate and squamoid cells were bland and positive for P63 and P40. Pulmonary adenocarcinoma and squamous cell carcinoma-associated cells usually show significant nuclear atypia and histological or immunohistochemical evidence of glandular or squamous differentiation. Adenocarcinoma cells usually stain positive for thyroid transcription factor-1 and napsin A, whereas squamous cell carcinoma cells are P63- and P40-positive. In contrast, ACC-associated cells are negative for all these markers. Renal clear cell carcinoma metastasis can be identified based on clinical history. The presence of PAX8 and CD10 markers can help identify its renal origin. Carcinoid tumors with clear or eosinophilic cell components usually stain positive for chromogranin A, synaptophysin, and CD56, while ACC stains negative for these markers. Mammary analogue secretory carcinomas can also occur in the lungs and are very similar to ACC in morphology, making it difficult to differentiate between the 2 types.^[27,28]^ However, mammary analogue secretory carcinoma of the lung was also observed to harbor *ETV6-NTRK3* rearrangements and was positive for mammaglobin and S-100, which cannot be detected in cases of ACC.^[27]^

Because ACC of the lung is rare, its definitive histopathological prognostic factors have not been elucidated. All 32 patients reported underwent surgery. Of those with documented follow-up, only 1 patient had tumor recurrence 20 months after surgery. Thus, ACC of the lung or trachea is classified as a low-grade tumor. Complete surgical resection of ACC of the lung or trachea is curative and feasible.

In summary, we report a case of primary tracheal ACC. Surgical resection is the primary treatment option for this type of tumor. Careful examination of histological features and immunohistochemistry is essential for correct diagnosis and prognosis evaluation.

## Author contributions

**Conceptualization:** Hong-Tao Xu.

**Methodology:** Zhi-Qiang Wang, Xiu-Feng Li, Li-Qian Chen, Hai-Ning Zhang, Ke-Xin Zhang.

**Funding acquisition:** Hong-Tao Xu.

**Investigation:** Mai-Qing Yang.

**Resources:** Hong-Tao Xu.

**Writing – original draft:** Mai-Qing Yang, Hong-Tao Xu.

**Writing – review & editing:** Mai-Qing Yang, Hong-Tao Xu.
